# Filtration of Gene Trees From 9,000 Exons, Introns, and UCEs Disentangles Conflicting Phylogenomic Relationships in Tree Frogs (Hylidae)

**DOI:** 10.1093/gbe/evad070

**Published:** 2023-05-02

**Authors:** Carl R Hutter, William Duellman

**Affiliations:** Biodiversity Institute and Department of Ecology and Evolutionary Biology, University of Kansas; Museum of Natural Sciences and Department of Biological Sciences, Louisiana State University; Biodiversity Institute and Department of Ecology and Evolutionary Biology, University of Kansas

**Keywords:** anura, exon capture, gene tree estimation error, target capture, UCEs

## Abstract

An emerging challenge in interpreting phylogenomic data sets is that concatenation and multi-species coalescent summary species tree approaches may produce conflicting results. Concatenation is problematic because it can strongly support an incorrect topology when incomplete lineage sorting (ILS) results in elevated gene-tree discordance. Conversely, summary species tree methods account for ILS to recover the correct topology, but these methods do not account for erroneous gene trees (“EGTs”) resulting from gene tree estimation error (GTEE). Third, site-based and full-likelihood methods promise to alleviate GTEE as these methods use the sequence data from alignments. To understand the impact of GTEE on species tree estimation in Hylidae tree frogs, we use an expansive data set of ∼9,000 exons, introns, and ultra-conserved elements and initially found conflict between all three types of analytical methods. We filtered EGTs using alignment metrics that could lead to GTEE (length, parsimony-informative sites, and missing data) and found that removing shorter, less informative alignments reconciled the conflict between concatenation and summary species tree methods with increased gene concordance, with the filtered topologies matching expected results from past studies. Contrarily, site-based and full-likelihood methods were mixed where one method was consistent with past studies and the other varied markedly. Critical to other studies, these results suggest a widespread conflation of ILS and GTEE, where EGTs rather than ILS are driving discordance. Finally, we apply these recommendations to an R package named PhyloConfigR, which facilitates phylogenetic software setup, summarizes alignments, and provides tools for filtering alignments and gene trees.

**Significance**

A major goal for systematic biologists—and evolutionary biologists in general—is understanding species relationships, as inferred from resolved, well-supported phylogenies. However, an emerging challenge in interpreting phylogenomic data sets is that different analytical approaches may produce conflicting phylogenetic results, and the trustworthiness between concatenation and multi-species coalescent species tree methods remains up for debate. We apply these ideas to Hylidae treefrogs using an expansive phylogenomic data set of ∼9,000 markers. We find conflicting topologies between concatenation and species tree methods; however, after filtering alignments and gene trees, we find that removing shorter, less informative alignments reconciled the conflict between concatenation and species tree methods with increased gene concordance. Importantly, we provide recommendations and solutions for interpreting phylogenomic results and suggestions for future study designs.

## Introduction

A major challenge in interpreting large phylogenomic data sets containing thousands of markers is that many phylogenetic relationships remain ambiguous because concatenation and species tree methods (i.e., summary species tree methods, site-based, and full-likelihood methods) often lead to conflicting phylogenetic results (Song et al. 2012; Gatesy and Springer 2014; Pyron et al. 2014; Giarla and Esselstyn 2015; Lambert et al. 2015; Chan et al. 2020a). It is intensely debated whether concatenation or species tree methods are more appropriate and reliable for analyzing large phylogenomic data sets (Gadagkar et al. 2005; Kubatko and Degnan 2007; Edwards 2009; Knowles 2009; Simmons and Gatesy 2015; Gatesy and Springer 2014; Edwards et al. 2016; Mallet et al. 2016). To a lesser extent, full-likelihood methods have been used for analyzing phylogenetic data, these methods typically are restricted to smaller data sets as they are computationally intensive (Yang 2015; Flouri et al. 2018). For very large phylogenomic data sets (>1,000 markers), approaches also differ in the type of data employed: Concatenation methods combine each individual alignment into a single alignment for phylogeny estimation whereas summary species tree methods use trees estimated from individual alignments (i.e., “gene trees”) to consider the genealogical history of each marker (Edwards 2009; Liu et al. 2009a, 2009b; Liu et al. 2010).

Before data sets contained thousands of markers, concatenation methods were predominantly used for estimating species trees by concatenating together a small number of markers that were easily analyzable with tree-building methods used at the time (Steel and Penny 2000; Edwards 2009). However, with the more recent ability to sequence thousands of markers, studies have shown that concatenation can lead to a topology different from the true species tree with strong support when there is high discordance among the individual gene trees (Rokas et al. 2003; Edwards et al. 2007; Song et al. 2012; Jarvis et al. 2014; Zhang et al. 2014; Crowl et al. 2017; Reddy et al. 2017; Chan et al. 2020a, 2020b). Discordance in the gene trees used to estimate species trees is broadly caused by natural processes such as incomplete lineage sorting (ILS), horizontal gene transfer, gene loss or duplication, natural selection on genes or sites, and hybridization (Maddison 1997; Edwards 2009; Liu et al. 2009a, 2099b; Knowles 2009; Hobolth et al. 2011; Knowles et al. 2018). Furthermore, methodological artifacts such as model inadequacy, short alignments, limited phylogenetic informativeness, or errors in sequence assembly or alignment can lead to gene tree estimation error or gene tree estimation error (GTEE) (Xi et al. 2012, 2015; Hahn and Nakhleh 2016; Blom et al. 2017; Reddy et al. 2017; Richards et al. 2018). Therefore, concatenation relies on the true species relationships being reflected in the sequence data although can be misled if there are high levels of homoplasy or noise among the gene trees (Townsend et al. 2012; Dornburg et al. 2019).

ILS, where genes in lineages from the same population fail to coalesce, and instead coalesce with lineages from a more distantly related population, may lead to species-tree inference errors if ILS is not considered (Hudson 1983; Tajima 1983; Degnan and Rosenberg 2006). A higher prevalence of ILS is expected when the species tree internal branches are short, which increases the chances of a branch coalescing with a non-sister branch (Degnan and Salter 2005; Degnan and Rosenberg 2006). Studies show that concatenation analyses estimate strongly supported although misleading topologies when there are high levels of gene discordance from ILS (Kubatko and Degnan 2007; Gatesy and Springer 2014; Linkem et al. 2016; Pollard et al. 2006; Chan et al. 2020a). To account for ILS, researchers have developed new analytical methods by modeling the multispecies coalescent and explicitly accounting for gene tree-species discordance (Edwards 2009; Liu et al. 2009a, 2009b; Nakhleh 2013; Gatesy and Springer 2014; Xu and Yang 2016).

Gene tree estimation error resulting in discordant gene trees (erroneous gene trees: “EGTs”) could be an important source of error driving discordance between summary species tree methods and concatenation. An often overlooked and important assumption of summary species tree methods is that gene trees are error free, whereby most gene tree variation is attributed to ILS (Edwards et al. 2007; Edwards 2009; Knowles 2009; Liu et al. 2009a, 2009b; Hobolth et al. 2011). In practice, however, it is likely that there is abundant GTEE resulting from poor model-fit, short alignment lengths, low levels of phylogenetic informativeness, or even the type of phylogenetic marker used (Hahn and Nakhleh 2016; Xi et al. 2015; Blom et al. 2017; Reddy et al. 2017; Richards et al. 2018; Burbrink et al. 2020). Past studies have shown that GTEEs resulting in abundant EGTs are problematic for species tree methods using gene trees as an input and have demonstrated that filtering out gene trees with high GTEE can improve support (Gatesy and Springer 2014; Roch and Warnow 2015; Xi et al. 2015; Springer and Gatesy 2016; Molloy and Warnow 2018; Bossert et al. 2021; Cai et al. 2021). When there are many EGTs, erroneous histories resulting from GTEE could be supported when they are common enough.

A potential solution is to combine shorter alignments into larger alignments (“statistical binning”; Bayzid et al. 2015) or to eliminate shorter alignments by filtering prior to gene tree estimation for features that could affect error (i.e., length, parsimony-informative sites (PISs), and missing data). The statistical binning approach whereas promising has been shown to give misleading results (Streicher et al. 2018), with one study finding that statistical binning leads to model violation from combining markers with different coalescent histories, where 92% of their binned markers were found to be composed of multiple coalescent histories (Adams and Castoe 2019). A promising alternative to binning is filtering, which is commonly done in ultra-conserved element (UCE) studies to improve support for relationships and remove low information UCEs (e.g., Doyle et al. 2015; Hosner et al. 2016; Branstetter et al. 2017; Gilbert et al. 2018; Molloy and Warnow 2018; Mclean et al. 2019). Conversely, in a simulation study, Molloy and Warnow (2018) filtered gene trees using a limited set of criteria (missing data and phylogenetic signal) and found that filtering improved the accuracy of the summary species tree methods when levels of ILS were low to moderate, and GTEE was high, which was a condition rarely encountered in their simulations. Despite the absence of such simulation conditions, empirical studies could potentially more commonly have low levels of ILS and high GTEE. It remains unknown how common these conditions are in empirical data sets, and analysis of method performance offers an important complement and comparison to simulation studies.

Marker selection is an increasingly important issue for phylogenomic studies as marker types may vary in the degree of GTEE. UCEs and exonic markers are the most common types employed and have resolved previously ambiguous relationships across the tree of life (Decker et al. 2009; Crawford et al. 2012; Faircloth et al. 2012, 2013; McCormack et al. 2013; Brandley et al. 2015; Smith et al. 2014; Hugall et al. 2016; Mitchell et al. 2017; Bragg et al. 2018; Streicher et al. 2018). Prior to the wide availability of expansive phylogenomic data sets, noncoding intronic sequences promised the potential to resolve problematic nodes because they are faster-evolving and thus more informative at shallow phylogenetic scales, although could prove problematic on larger scales (Armstrong et al. 2001; DeBry and Seshadri 2001; Krauss et al. 2008; Allen and Omland 2003; Folk et al. 2015). Recently, analysis of intronic sequence has increased in phylogenomic studies, although the results have been mixed compared with other data types (Townsend 2007; Fischer and Steel 2009; Townsend et al. 2012; McCormack et al. 2013; Folk et al. 2015; Prum et al. 2015; Chen et al. 2017; Reddy et al. 2017; Dornburg et al. 2019). Therefore, selecting the best combination of molecular markers for phylogenetic studies remains a fundamental challenge; with numerous studies comparing the performance and phylogenetic incongruence among marker types with no clear ideal solution (Fong and Fujita 2011; Hong-Wa and Besnard 2013; Gilbert et al. 2015; Chen et al. 2017; Jarvis et al. 2014; Karin et al. 2020; Cloutier et al. 2019; Chan et al. 2020a, 2020b).

**Fig. 1:**
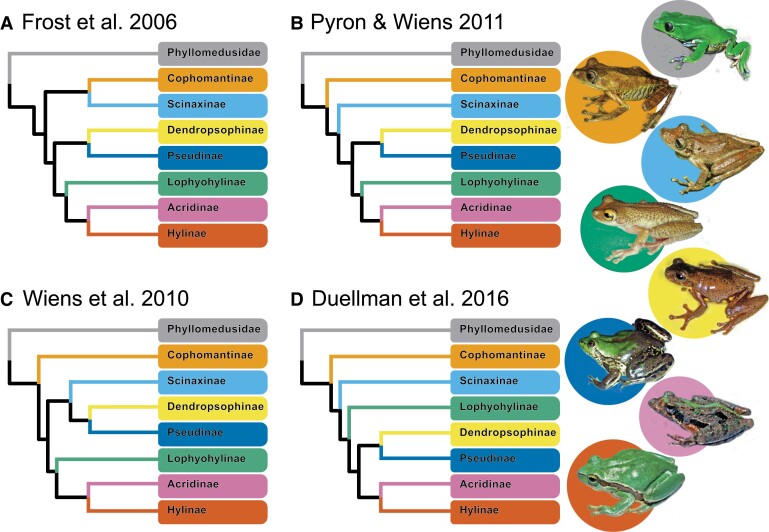
The history of major studies in Hylidae tree frogs is illustrated below. The clades are shown by subfamily and the representative frog photographs are ordered by the clade in the phylogeny (credit: W. Duellman).

To understand how GTEE leads to conflicting relationships among phylogenetic tree-building methods, we examine phylogenetic relationships in “hylid” tree frogs (collectively “Arboranae”), specifically of the family Hylidae. Hylids are among the most charismatic and species-rich frog families, representing ∼15% of the world's frogs (AmphibiaWeb 2022). This clade has received substantial attention from systematists, resulting in an active taxonomic history. The first molecular studies of the Hylidae divide them into three subfamilies: Hylinae, Phyllomedusinae, and Pelodryadinae (Wiens et al. 2005; Frost et al. 2006). These subfamilies remained monophyletic in later molecular studies; however, the number of genera and other taxonomic units was often revised, and phylogenetic relationships often received low support (Wiens et al. 2010; Pyron and Wiens 2011). Recently, this group of frogs has been categorized into three families: Hylidae, Phyllomedusidae, and Pelodryadidae to help manage an increasing number of species (Duellman et al. 2016). Within the revised Hylidae, seven subfamilies were named: Acrisinae, Hylinae (Holarctic and Middle American), Pseudinae, Dendropsophinae, Lophyohylinae, Scinaxinae, and Cophomantinae. However, uncertainty remains whether these groups are natural as they have had poor support for their monophyly in past studies (Duellman et al. 2016; Faivovich et al. 2018). Furthermore, the interrelationships among subfamilies have remained ambiguous across numerous studies (fig. 1; Wiens et al. 2005; Frost et al. 2006; Wiens et al. 2010; Pyron and Wiens 2011; Duellman et al. 2016).

We aim to disentangle the phylogenetic relationships of hylid tree frogs with a new and expansive phylogenomic data set comprising different molecular marker types (FrogCap; Hutter et al. 2022), which targets ∼2,300 UCEs and ∼6,000 exons, along with their flanking intronic regions. We provide the first phylogenomic analysis of tree frogs from the family Hylidae and compare exons, introns, and UCEs for their ability to provide support for phylogenetic relationships. We predict that subfamilies named in Duellman et al. (2016) are monophyletic, where these clades were also monophyletic in past studies although with low support (fig. 1). We also make available a new R package named PhyloConfigR, which can create setup files for popular phylogenetic software (BPP, ASTRAL-III, and SVDQuartets), summarize statistics across alignments, filter alignments and gene trees by various alignment statistics, and concatenate alignments all within R. Using PhyloConfigR, we filter potential EGTs using alignment metrics that could lead to GTEE: alignment length, number of PIS, alignment proportion of PIS, and missing data from proportion of species missing from an alignment. Finally, we address the question of whether expensive and large phylogenomic data sets provide different and more strongly supported results than existing archived GenBank data and discuss the lasting importance of these data to emerging research communities where funding access is often limited.

## Results

### Sequence Capture Evaluation

We sequenced 26 samples using the FrogCap probe-set, which totaled 29,321 mega base pairs (Mbp) of raw sequence data for these samples (sample raw read statistics included in supplementary tables s1 and S2, Supplementary Material online). The mean base pair yield across samples was 1,127.7 ± 518.4 (range: 419.4–2,903.4) Mbp. The mean number of raw reads per sample was 7,468,509 ± 3,432,832 (range: 2,777,240–19,228,002) reads. Raw reads were filtered to remove exact duplicates, low complexity, and poor-quality bases, adapter, and contamination from other nontarget organisms, which resulted in a mean 77.1% ± 18.2% of reads (range: 10.6–91.0%) passing the quality filtration steps (mean: 834.9 ± 436.1 Mbp; range: 117.3–2,242.5 Mbp). After merging paired-end reads and reducing redundancy (removing duplicate and completely overlapping reads), there was a mean 347,585 ± 142,315 (range: 87,772–755,347) merged paired-end reads (and singletons) used as input for assembly (supplementary table S3, Supplementary Material online). After assembly, the samples yielded a mean of 17,930 ± 6,579.2 (range: 5,378–37,957) contigs, which had a mean length of 867.3 ± 43.6 (range: 128–22,652) bp (supplementary table S4, Supplementary Material online).

### Alignment Summary

Alignment and quality control of the multiple sequence alignments prior to trimming results in 8,761 total aligned markers, with a mean 19.0 ± 6.2 (range: 3–26) samples per alignment (table 1), which remained consistent across data sets (supplementary fig. S1, Supplementary Material online). Sample occupancy derived from the sequence alignments was also high across samples, with most samples having greater than 6,000 aligned markers (supplementary fig. S1, Supplementary Material online). The Unified data set (where all markers are included, and introns are not trimmed from exons) has a mean 1,960.5 ± 1,024.2 (range: 271–18,866) bp per alignment, totaling 17,176,214 bp.

**Table 1:** Each Marker Type Is Summarized, After Trimming and Processing

	Unified	Exons	Introns	UCEs	Genes
Alignments	8,679	4,328	4,197	2,762	1,599
Samples	18.7 ± 6.3 (5–26)	17.7 ± 6.4 (5–26)	17.5 ± 6.1 (5–26)	20.1 ± 6 (5–26)	19.6 ± 6 (5–26)
Total base-pairs	4,319,594	1,179,159	1,595,447	1,857,575	653,580
Informative sites (bp)	1,280,180	265,936	804,869	414,221	149,213
Informative sites (%)	30.4 ± 13.6 (0–70.13)	21.4 ± 8.9 (0–75.4)	52.3 ± 21.1 (0.56–96)	21.4 ± 11.2 (0–73.2)	22 ± 7.5 (0–46.46)
Alignment length (bp)	497.7 ± 303.9 (94–5,840)	272.4 ± 335.5 (78–5,064)	380.1 ± 177.4 (85–2,296)	672.5 ± 280.3 (94–2,700)	408.7 ± 416.0 (84–5,478)
Alignment length (bins)					
100–200 bp	268	2,230	670	72	455
201–500 bp	5,112	1,770	2,930	865	797
501–1,000 bp	3,067	218	754	1,510	266
1,001–2,000 bp	201	82	23	338	65
2,001–6,000 bp	38	28	1	2	16

After separating the intron and exon sequences from the aligned set of contigs and trimming, the Exon data set had 4,328 alignments totaling 1,179,159 bp, and the Intron data set containing only noncoding flanking sequence from both ends of the exons had 4,197 joined intron alignments totaling 1,595,447 bp of sequence data. Additionally, the Exon data set had a mean 272.5 ± 335.5 (range: 100–5,064) bp per alignment, whereas the Intron data set has a mean 380.1 ± 177.4 (range: 100–2,296) bp per alignment. Multiple sequence alignments for the UCE data set had 2,762 aligned UCEs totaling 1,857,575 bp of data after filtration and trimming. The UCE data set had a mean 672.6 ± 280.3 (range: 100–2,700) bp per alignment. The final set of alignments for the FrogCap data concatenated individual exons from the same gene (the Gene data set), which resulted in 1,599 gene alignments totaling 653,580 bp. The Gene data set had a mean 408.7 ± 416.0 (range: 100–5,478) bp per alignment (supplementary fig. S2, Supplementary Material online).

### Phylogenetics

**Fig. 2:**
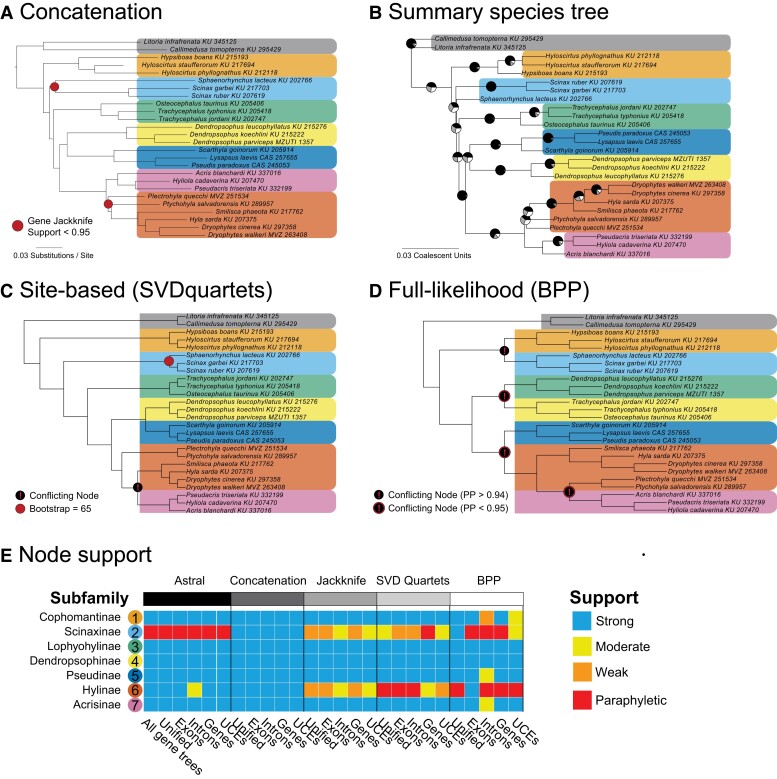
Phylogenetic relationships and support for Hylidae subfamily relationship from the unfiltered data set for concatenation-based and minimum-coalescent species tree analyses. All concatenation-based analyses agreed with the topology shown in (*A*), mostly with strong support. The branch lengths are from the 70% taxon-sampled matrix analysis, and the red dots at nodes are analyses with high gene-jackknife support (i.e., at least 95% of replicates have the shown topology). The results from the species tree analyses were mixed, where the ASTRAL-III results are shown in (*B*). The topology in (*B*) is from the Unified (exons + flanking introns) ASTRAL-III analysis, where pie charts at branches represent the quartet-score frequency at that branch (all nodes had strong PP support). In (*C*), the site-based SVDquartets Unified data set tree is shown, which had one poorly supported node and one conflicting node. In (*D*), the full-likelihood BPP Unified data set is shown, which had several conflicting nodes, most with poor support. In (*C*) and (*D*), the branch lengths are equal by using a cladogram. Colors indicate each focal subfamily (see fig 1) that are assessed for support in (*D*). In (*E*), the node support is shown for each focal subfamily across the different data types and analyses.

We found that the concatenation analyses strongly supported all focal subfamilies in all types of markers with strong support (figs. 2*A* and S3, Supplementary Material online); however, gene jackknifing gave low support for the monophyly of Scinaxinae and Hylinae, suggesting conflicting signals or GTEE when resampling genes (supplementary fig. S4, Supplementary Material online). To account for ILS, we used the gene trees to estimate the topology using ASTRAL-III and found that most subfamilies were monophyletic in all analyses generally with strong support, except Scinaxinae and Hylinae (figs. 2*B* and S5, Supplementary Material online). Scinaxinae was always non-monophyletic in ASTRAL-III trees (fig. 2*B*), where the genus *Sphaenorhynchus* was not sister to *Scinax*, although shifted around the backbone of Hylidae. Conversely, Hylinae was monophyletic in all ASTRAL-III analyses with strong support except with the Intron data set. Next, we used SVDquartets, a site-based species tree method, with the aim to alleviate GTEE by using the underlying sequence data. Scinaxinae was recovered as monophyletic in most SVDquartets analyses except the Gene data set, albeit with low to moderate support (fig. 2*C*). Hylinae was non-monophyletic in many of the SVDquartets analyses because the relationships for *Plectrohyla* and *Ptychohlya* would cause Acrisinae to be nested within Hylinae (supplementary fig. S6, Supplementary Material online). Finally, we used BPP, a full-likelihood site-based species tree method, which like SVDquartets could potentially alleviate GTEE by using the underlying sequence data. The results for BPP received the lowest collective posterior probability support where some subfamilies strongly supported in all prior analyses were poorly supported here (figs. 2*D* and *E* and S7, Supplementary Material online). This is likely due to computational tractability from the large number of markers, where the BPP analyses received poor mixing and did not visit enough distinct trees. The number of distinct trees for each data set was: 1) Unified, run 1 = 3, run 2 = 3; 2) Exon, run 1 = 6, run 2 = 5; 3) Intron, run 1 = 7, run 2 = 7; 4) UCE, run 1 = 120, run 2 = 114; and 5) Gene, run 1 = 295, run 2 = 310.

When assessing results by marker type across analytical methods, we find several patterns. First, the Unified data set, which is the collection of all the captured markers without trimming the flanking region such that each individual marker has more base pairs than other data types, performed the best across all analyses. The Gene data set, where exons from the same gene were binned together, did not perform as well as expected given the length of the alignments and often had Scinaxinae and Hylinae as paraphyletic. Exons and UCEs performed similarly with some analyses recovering Scinaxinae and Hylinae as monophyletic. Finally, the Intron data set performed the worst, having the lowest support and highest frequency of paraphyletic subfamilies (fig. 2*E*).

### Branch Lengths and Support

**Fig. 3:**
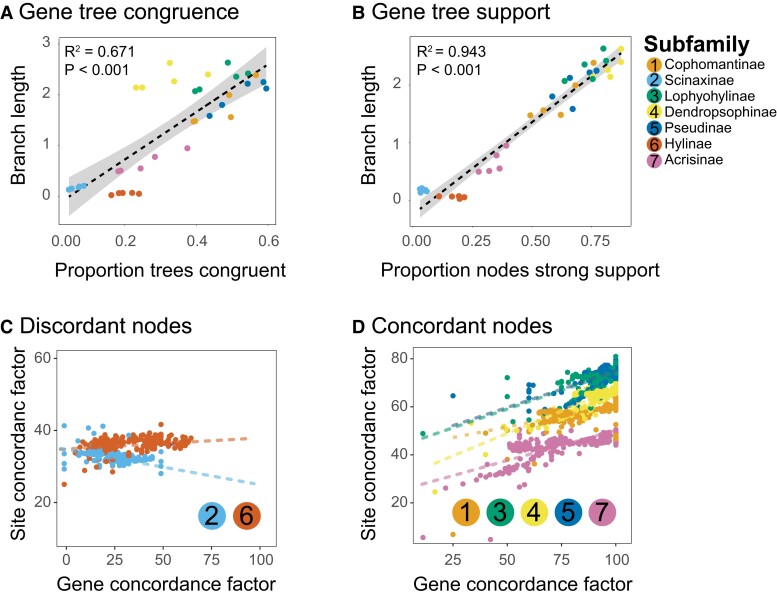
Relationship between branch lengths (from the ML concatenation trees estimated from IQ-Tree; number of nucleotide substitutions per site) and support (bootstrap support from IQ-Tree), and the impact of filtering on gene and site concordance factors (gCF and sCF) for each of the subfamily relationships. In (*A*), a significant positive relationship is shown between branch lengths and the proportion of gene trees that support the monophyly of subfamilies. (*B*) shows a strong and significant relationship between branch lengths and the proportion of subfamily nodes that are strongly supported (>95 bootstrap). In (*C*) and (*D*), gCF and sCF are computed from filtration data sets across focal subfamily nodes. In (*C*), the nodes for Scinaxinae and Hylinae have an inverse or no relationship between gCF and sCF, revealing that the conflicting genes and sites result in EGTs that contribute to discordance. Conversely, in (*D*), the nodes concordant across most filtration replicates have a positive relationship between gCF and sCF, suggesting that the sites in alignments for gene trees at these nodes are consistent with the gene tree topology. ML, maximum likelihood.

We assessed whether short branch lengths are responsible for phylogenetic incongruence. In our first analysis, we found a significant positive relationship between branch lengths and the proportion of gene trees that are monophyletic for each subfamily within each analysis (fig. 3*A*). This result suggests that shorter branches are associated with clades that are often not monophyletic in the gene trees. In our second analysis, we also found a significant positive relationship between branch length and the proportion of gene trees that strongly support (90 bootstrap or greater) the monophyly of subfamilies (fig. 3*B*), suggesting that shorter branch lengths are indeed related to poor support among gene trees, which is consistent with theoretical predictions (Fischer and Steel 2009; Townsend and Leuenberger 2011; Townsend et al. 2012; Su and Townsend 2015; Steel and Leuenberger 2017; Dornburg et al. 2019).

### Filtration

**Fig. 4:**
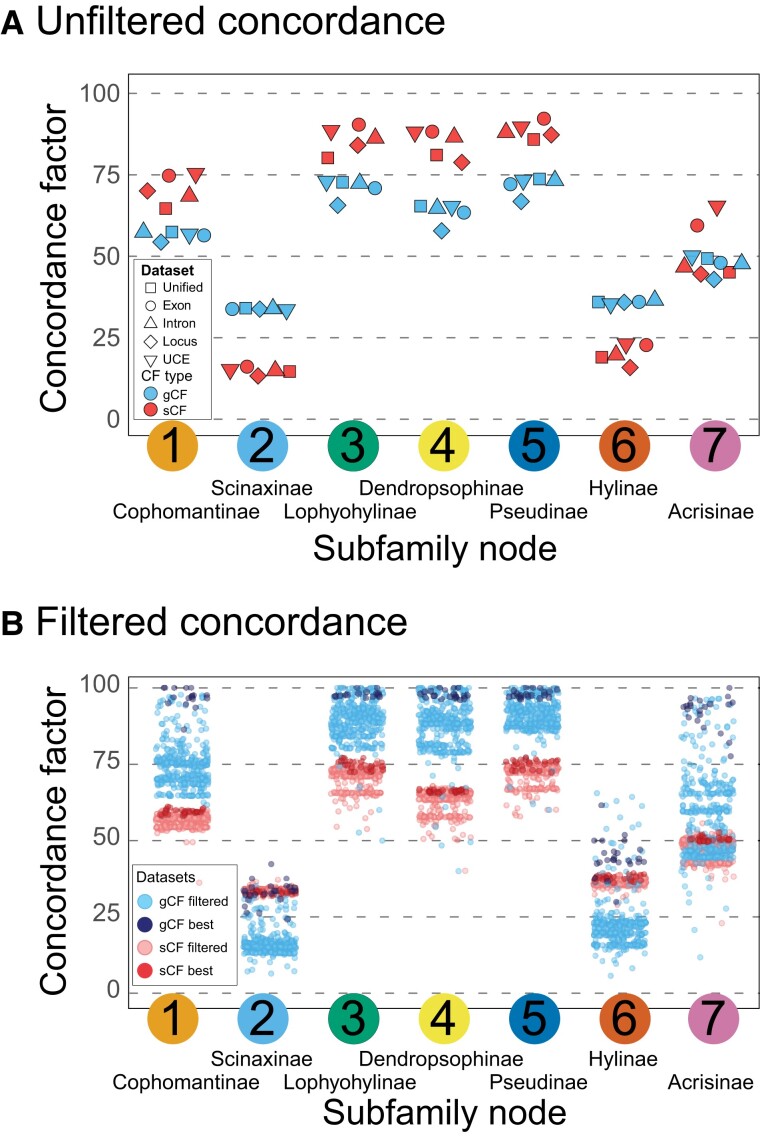
Concordance factors for unfiltered and filtered data sets are illustrated. When assessing concordance across the unfiltered data sets in (*A*), we show the gCF and sCF (gene and site concordance factors) support from the different marker types for each subfamily clade. The conflicting nodes of Scinaxinae and Hylinae have an inverse relationship between gCF and sCF (higher gCF, lower sCF instead of the opposite). In (*B*), the concordance factors for filtered data sets are plotted, where the filtered data sets have increasing support from gCF and sCF under some filtration schemes.

**Fig. 5:**
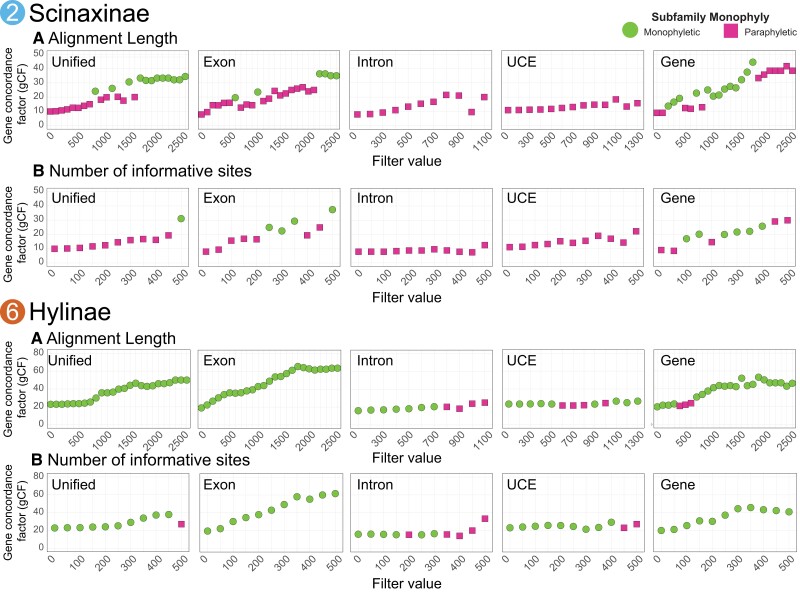
Gene and alignment filtering phylogenetic results across the Unified, Exons, Introns, UCEs, and Genes (i.e., binned exons) data sets where the concordance factors (gene and site; gCF and sCF) are calculated for each filtration replicate for the five data sets. The results show that filtering leads to monophyly for the traditional subfamily relationships and that longer alignment types (Unified and binned genes) perform better than other data types. The filters used were: (*A*) the number of PIS in an alignment; and (*B*) the base-pair length of the alignment. Additional filtering plots are shown in the Supplementary material (proportion of sampled taxa and proportion of PIS). The dot plots for each filter depict the effect filtering has on the gene concordance factor (gCF), where the pink squares indicate filtered trees where the subfamily is paraphyletic, and green shows filtered trees where the focal subfamily is monophyletic. The “2” and “6” represent the node numbers introduced in figure 2. Results using sCF are in the Supplementary material. sCF, site concordance factor; PIS, parsimony-informative site.

To assess whether filtering EGTs can improve summary species tree estimates, we filtered gene trees prior to species tree estimation. Our results show that alignment-based filtration of gene trees generally leads to higher support in gene and site concordance factors (gCF and sCF) when compared with the unfiltered data sets (fig. 4). When assessing filtration replicates individually, there is a gradual improvement in support and subfamily monophyly in the filtered summary species trees supporting our predictions (fig. 5). Filtering for alignment length was the most successful (fig. 5*A*), where low filtering brought monophyly in Hylinae and moderate filtration found Scinaxinae as monophyletic. In addition, the number of PIS was successful in two of the high-value filtrations for Scinaxinae and was more successful in Hylinae, where moderate-to-low filtration alleviated conflict surrounding this clade (fig. 5*B*). Filtering alignments for taxon sampling and proportion of PIS had little impact on support (supplementary figs. S8–S11, Supplementary Material online). Most significantly, in most filtered data sets, the new species topology matched the expected subfamily monophyly from past studies, strongly supporting the previously non-monophyletic or poorly supported subfamilies Scinaxinae and Hylinae (fig. 5). Scinaxinae previously was paraphyletic in all species tree analyses, and filtration by alignment length has led to monophyly and much higher concordance factors support and matches the concatenation topology (fig. 5). Hylinae is now strongly supported from filtering alignment length and number of PIS for the concatenation topology, with a substantial increase in concordance factors (fig. 5).

Unexpectedly, when assessing the filtration results together (IQ-Tree, SVDquartets, and ASTRAL-III), we find that the topology from species trees is concordant with the topology from concatenation, which matches the results of past studies, but these studies had poor support (fig. 1). The Unified and Gene data sets were the most strongly supported and supported subfamily monophyly whereas the Intron and UCE data sets did not recover Scinaxinae as monophyletic in any data set (fig. 5). In addition, we also found that filtration had little effect on the results of IQ-Tree (supplementary fig. S8, Supplementary Material online), where very high levels of filtration decreased support for the concatenation topology. In the filtered ASTRAL-III analyses, increased filtration of alignment length mostly supported the concatenation topology and increased CF support for these relationships (supplementary fig. S9, Supplementary Material online).

Finally, SVDquartets performed similarly well in the filtration analyses. For Hylinae, filtering by the number of PIS and alignment length remedied the paraphyly found in the unfiltered data sets (supplementary figs. S10–S11, Supplementary Material online), which is concordant with the concatenation and summary species tree results. In addition, Scinaxinae received stronger support with moderate filtration; however, high levels of filtration led to poorly supported and incongruent results at other nodes (supplementary figs. S10 and S11, Supplementary Material online). None of the filtered data sets with complete subfamily monophyly had congruent relationships with the SVDquartets analysis counterpart.

## Discussion

We sequenced a new and expansive data set of ∼9,000 markers from exons, introns, and UCEs for the frog family Hylidae and compared the results from concatenation and species tree analytical methods to assess the impact of GTEE on subfamily monophyly and phylogenetic support. Our results initially strongly support the monophyly of most of the subfamily clades. Despite the massive increase in genetic data from prior studies, we initially found conflicting results among analytical methods for Scinaxinae and Hylinae. Concatenation and species tree methods estimated different topologies (fig. 2); however, concatenation provided strong support whereas the species trees summary methods were not strongly supported (fig. 2*A* and *B*). ASTRAL-III and SVDquartets estimated paraphyletic relationships for the subfamily Scinaxinae across all data types, and Hylinae was paraphyletic in some SVDquartets analyses (fig. 2*C*). Using the likelihood-based species tree method BPP, we found that a data set of this size with many thousands of markers was computationally intractable, and the results received poor mixing and a small number of trees visited despite running the software for several weeks. We also demonstrate that shorter branch lengths from concatenation have lower support values (fig. 3*A* and *B*), which may in part explain the uncertainty surrounding these nodes. Despite these initially conflicting results, we found that filtering out gene trees that had few informative sites or were short in length resulted in most summary species tree analyses finding these subfamilies monophyletic with strong support, which matched the concatenation results.

### Hylidae Relationships

Our phylogenomic study was initially able to provide strong statistical support for the monophyly of most Hylidae subfamilies as named in Duellman et al. (2016) that were previously not well-supported (figs. 1*D*, 2, and 3). After filtering short, low information alignments and their corresponding gene trees, we were able to reconcile the conflict between concatenation and species tree methods in topology (figs. 4 and 5). Whereas some uncertainty remains in the middling posterior probabilities and relatively low gene and site concordance for the Scinaxinae subfamily, filtering led to species tree methods estimating the concatenation topology and increase gene concordance by 4-fold. For the subfamily Hylinae, which had conflict among analytical methods and data types with low posterior probability support, filtering was even more effective, leading to strong posterior probability (PP) support and gene concordance (fig. 5). Despite our abundance of markers, filtered data sets that supported Scinaxinae had relatively few markers (25–200), so it is possible that an increase in longer and more informative alignments could eventually definitively support this subfamily in future studies.

Apart from the subfamily monophyly, the final shared topology from concatenation and species tree methods has been supported traditionally on smaller multi-locus Sanger studies (e.g., Duellman et al. 2016; Faivovich et al. 2018). Our final filtered topologies most closely aligned with Duellman et al. (2016) and was identical in overall relationships among subfamilies. In addition, Faivovich et al. (2018) found the same general relationships that we recovered, except with increased taxon sampling they found that genera not included in this study were paraphyletic (*Ptychohyla* and *Duellmanohyla*). Our study conflicted with other recent past studies, for example in Pyron and Wiens (2011), the phylogenetic position of Lophyohylinae swapped positions with the Dendropsophinae + Pseudinae clade. In addition, in Wiens et al. (2010), Scinaxinae was found sister to Dendropsophinae + Pseudinae. This study lays the groundwork for future phylogenetic research in the family Hylidae, and hypotheses regarding relationships among the dozens of other genera within subfamilies remain to be tested.

### Alignment-Based Gene Tree Filtering

We demonstrate that alignment-based gene tree filtering of EGTs can lead to reconciliation between concatenation and summary species trees methods. Our results support our predictions that filtering possible EGTs with shorter alignment lengths and fewer PIS leads to more consistent topologies and stronger support. We tested this prediction by filtering gene tree data sets using alignment statistics (sampling, alignment length, proportion, and number PIS) and estimated new trees using IQ-Tree, ASTRAL-III, and SVDquartets. We found that filtering by taxon sampling (or missing data) and PIS proportions provided little improvement in topology and support (supplementary figs. S8–S11, Supplementary Material online). In contrast, the number of PIS and alignment length had substantial impacts on the topology matching it close to expectations and increased support, supporting our predictions (fig. 5). Importantly, we find that with longer, more informative alignments, the concatenation (fig. 2*A*) relationships match the summary species tree topology after gene tree filtering (fig. 5). We note that simulation studies found similar results (Roch and Warnow 2015; Molloy and Warnow 2018), in that filtering out gene trees with high amounts of GTEE results in stronger support in species tree methods under certain conditions (ILS is low and GTEE high), but this study did not assess specific alignment characteristics to determine how to best filter alignments in empirical systems. We consider that empirical validation is an important compliment to simulation studies, as rare model conditions could potentially be more common than presented in the model.

Significant to other phylogenomic studies, we demonstrate that violating the assumption of error-free gene trees in summary species tree methods can consistently lead to incorrect species trees, and unexpectedly, as a result, we found that the original concatenation tree had the “correct” topology (i.e., “correct” being that subfamilies are monophyletic like in most past studies). When filtering out alignments that are uninformative, we found that the summary species tree methods estimated new topologies consistent with the concatenation results, which suggests that relationships are not discordant because of ILS. Instead, we show EGTs that arise from misleading alignments are responsible for discordance, otherwise the concatenation result would be different. Interestingly, the site-based method SVDquartets (the full-likelihood BPP could not be compared because of computational tractability) also improved in the same ways by filtering out these alignments For Hylinae (but not Scinaxinae), despite being a site-based method. An explanation for this pattern is whereas shorter alignments may not have enough information for estimating gene trees, perhaps the phylogenetic signal per site in the short alignments is weak.

Additionally, whereas we show that the UCEs and Introns data sets were problematic for subfamily monophyly, and the Exon and Gene data sets were often successful in having the expected monophyly in subfamilies. The best performing for our data set is the Unified data set, which includes the target marker and any adjacent sequence bycatch (i.e., introns and UCE flanks), and generally has longer alignments than the other data types. However, these results are only generalizable to phylogenetic scales like the present study (i.e., family level), as marker utility varies based on phylogenetic scale (Su and Townsend 2015; Dornburg et al. 2019). We recommend that future studies can resolve many of these issues by using longer alignments from exons or genes (from concatenated exons) or even by concatenating UCEs from the same gene if available.

Finally, whereas alignment-based filtration of gene trees was successful in this present study, we suggest that researchers be mindful of potential pitfalls. First, researchers should be careful to test a variety of filtration parameters such that they do not unconsciously bias their results by selecting criteria that lead to trees that agree with their expectations. Second, the filtration values and criteria are data set specific, as some data sets may have a completely different number of alignments and parameters describing those alignments. As mentioned above, UCE data sets tend to have much shorter alignments (<1,000 bp), and filtration by large alignment lengths would be harmful and instead better filtration parameters might be centered on PIS. In addition, filtration could have unintended negative consequences such as entire taxa themselves being filtered out if they have substantial missing data or the final data set could end up with few PIS for phylogenomic analyses. In addition, if the group has ILS, filtration could be a net negative as ILS is modeled in species tree methods, and removal of markers with ILS could result in poor species tree estimation. Finally, filtering out alignments can bias other aspects of phylogenomic analyses; for example, filtering out slow evolving markers can impact branch lengths and divergence times, especially in likelihood-based coalescent methods where population sizes (theta) and divergence times (tau) would be impacted.

### Concordance Factors

We demonstrate the utility of gCF and sCF and provide examples of new situations where concordance factors provide additional insights on understanding gene-tree discordance in phylogenomic studies. Concordance factors can generally be used as a complimentary support metric that can be used alongside posterior probabilities or bootstrap support, because it can provide the relative proportions of gene trees and PIS from all alignments that support each branch in each topology (Minh et al. 2020). Using concordance factors, we find that species trees for the two initially non-monophyletic subfamilies (Scinaxinae and Hylinae) have an inverse relationship between gCF and sCF (fig. 3*C*), which could be explained by fewer gene trees supporting the subfamily monophyly but the sites within these genes collectively providing stronger support. Conversely, concordant subfamilies have a positive relationship between gCF and sCF (fig. 3*D*). After filtration, however, there is a substantial increase in gCF overall where better estimated gene trees result in more support from gCF for a species tree that matches the expected monophyly of subfamilies (fig. 5). We hypothesize the inverse relationship between genes and sites occurred because more sites supported the concatenation topology (which is why concatenation gave the correct topology prior to filtering), whereas many genes before filtration were EGTs biasing gene support.

In addition, we show that the filtration of gene trees has several other benefits by providing increased support measured through concordance factors and quartet score frequencies. Our results indicate that the number of PIS and alignment length had substantial impacts on the topology and support, where alignments with longer and more PIS provided higher quality gene trees (fig. 5). We find that filtering for longer, more informative alignments lead to increased gene and site concordance factors, often quadrupling the concordance factors from the unfiltered alignments. However, we found that filtering by taxon sampling and PIS proportions had no impact on the support and topology using concatenation; Scinaxinae remained paraphyletic in all ASTRAL-III analyses, and support was slightly higher for Hylinae in the ASTRAL-III intron data set (supplementary figs. S8–S11, Supplementary Material online). The explanation for why the proportion of PIS was not effective in filtration may be because short alignments have a high proportion of PIS but not enough of them have a consistent signal; our results support this by showing that longer alignments with higher counts of PIS are more important for increasing support. Together, these results underscore the importance of having longer and more informative alignments for phylogenetic analyses.

### Reconciliation of Concatenation and Species Tree Methods

The conflict between concatenation and species tree methods is widespread across many studies and our results offer some valuable recommendations and analytical tools to implement and achieve reconciliation through alignment-based gene tree filtering in future phylogenomic studies. When alignments have a short length or few PIS, EGTs could be confused for ILS which is problematic when species tree methods are designed to take ILS into account, and gene-tree uncertainty is ignored or not properly modeled, thus sources of error from EGTs will remain unaccounted. We show that only collections of gene trees from the longer alignments and with more PIS can greatly improve concordance factor support and expected subfamily monophyly (fig. 5); however, the level of filtration necessary varied for each node of interest (i.e., Scinaxinae vs. Hylinae). The amount of filtration needed may depend on underlying informative site support for that node as well as any background ILS that could also be obscuring the true relationship (Gilbert et al. 2018; Dornburg et al. 2019; Cai et al. 2021).

## Conclusions

These results raise an important consideration for systematic studies and increasing research capacity globally and in emerging economies: Will adding thousands of markers through expensive sequence capture studies reveal results that are significantly different from past studies? Our results initially found strong support for the monophyly of most of the subfamily clades except Scinaxinae and Hylinae. Despite the massive increase in genetic data from prior studies, we initially found conflicting results among analytical methods for Scinaxinae and Hylinae, and through alignment-based filtration, we were able to provide monophyly for Scinaxinae and Hylinae and strong support in some analyses. The BPP analyses were particularity problematic, which due to computational tractability from the large number of markers, the results had poor mixing and a small number of trees visited. As this was beyond the scope of this work, we recommend that future studies assess BPP in this group through a carefully designed study where markers are subsampled in smaller subsets and compared. Our filtering analyses indicate improved support and the expected topology with as few as 20 gene trees via ASTRAL-III, but only if the underlying alignments are informative enough to provide well-resolved and strongly supported relationships. Our results are also consistent with past Sanger sequencing studies that found the same topology using 10 markers, but many nodes were weakly supported (Duellman et al. 2016). We consider that this is possible because GTEE rather than ILS led to GTEE, and filtering out poor-quality markers can mitigate the negative effects of GTEE, but in cases of true ILS, more markers would be needed (Molloy and Warnow 2018; Dornburg et al. 2019). Researchers with limited funds could therefore target fewer but longer and more informative markers in future scaled-down probe set designs, such as the long markers used here, or the RELEC set of markers for other organisms (Karin et al. 2020). The number of markers may be an important consideration for researchers with limited access to research funding, especially in developing countries. By selecting fewer markers by which to invest probes, researchers could multiplex many times more samples because of lesser sequencing effort from the fewer target markers and increased sequencing capacity of newer Illumina platforms (386 or more unique index combinations have recently become available through Adapterama; Glenn et al. 2019; Bayona-Vásquez et al. 2019).

## Materials and Methods

### Taxon Sampling and DNA Extraction

To achieve phylogenetic representation across Hylidae, we selected three species (26 total samples) from each of the subfamilies: Acrisinae, Hylinae (Holarctic and Middle American), Pseudinae, Dendropsophinae, Lophyohylinae, Scinaxinae, and Cophomantinae. We also selected one species each from the families Phyllomedusidae and Pelodryadidae to be used as outgroups. The UCE data from 7 samples are first published in Portik et al., in press as part of a large UCE phylogeny of all frogs. Tissue samples for molecular work were obtained from the museum holdings of the University of Kansas (KU), California Academy of Science (CAS), Museum of Vertebrate Zoology at Berkeley (MVZ), and Museo de Zoología Universidad Technologica Indoamérica, Quito, Ecuador (MZUTI). Sample metadata are included as supplementary table S1, Supplementary Material online. Genomic DNA was extracted from the tissue samples with a PROMEGA Maxwell bead extraction robot. The resultant DNA was quantified using a PROMEGA Quantus Fluorometer. Approximately 500 ng total DNA was acquired and set to a volume of 50 μl through dilution with Promega elution buffer or concentration using a vacuum centrifuge when over 50 μl.

### Probe Design, Library Preparation, and Sequencing

Probe design was completed by Hutter et al. (2022) and is summarized here. Probes were designed by matching publicly available frog transcriptomes to genomes to find orthologous markers. Matching sequences were clustered by their genomic coordinates to detect presence/absence across species and to achieve full locus coverage. To narrow the locus selection to coding regions, each cluster was matched to available coding region annotations from the *Nanorana parkeri* genome (Sun et al. 2015). Markers from all matching species were then aligned using MAFFT (Katoh and Standley 2013) and had various statistics calculated to aid in marker selection. Additionally, 2,166 UCEs were selected from Streicher et al. (2018) and 86 markers previously used in Sanger sequencing (Feng et al. 2017) if they had at least 50% taxon sampling or greater, where the consensus sequence from each alignment after trimming was used to redesign probes for frogs. Finally, the selected markers were separated into 120-bp-long bait sequences with 2 × tiling (50% overlap among baits) using the MyBaits-2 kit with 120-mer sized baits. The selected markers also have an additional bait at each end extending into the intronic region to increase the coverage and capture of these areas. The baits were then filtered, keeping those: without sequence repeats, a GC content of 30–50%, and those that did not match their reverse complement or multiple genomic regions. Probes were synthesized as biotinylated RNA oligos in a MYBAITS kit with 40,040 baits (Arbor Biosciences, formerly MYcroarray Ann Arbor, MI).

The genomic libraries for the samples were prepared by the Arbor Biosciences library preparation service. Prior to library preparation, the genomic DNA samples were quantified with fluorescence using a Qubit and up to 4 μg was then sonicated with a QSonica Q800R instrument. After sonication and SPRI bead-based size-selection to modal lengths of roughly 300 bp, up to 500 ng of each sheared DNA sample was taken to Illumina Truseq-style sticky-end library preparation. Following adapter ligation and fill-in, each library was amplified for 6 cycles using unique combinations of i7 and i5 indexing primers, and then quantified using a Qubit. For each capture reaction, 125 ng of eight libraries were pooled, and subsequently enriched for targets using the MYbaits v 3.1 protocol. Following enrichment, library pools were amplified for 10 cycles using universal primers and subsequently pooled in equimolar amounts for sequencing. Samples were sequenced on an Illumina HiSeq 3000 with 150 bp paired-end reads.

### Data Processing and Alignment

A bioinformatics pipeline for filtering adapter contamination, assembling markers, and exporting alignments in different formats and data types is available at (bioinformatics-pipeline_stable-v1; https://github.com/chutter/FrogCap-Sequence-Capture). The pipeline is scripted in R statistical software (R Development Core Team 2018) using the BIOCONDUCTOR suite of packages (Ramos et al. 2017). The pipeline first cleans the raw reads of adapter contamination, low complexity sequences, and other sequencing artifacts using the program FASTP (default settings; Chen et al. 2018). Adapter-cleaned reads are next matched to a database of publicly available genomes from bacteria, invertebrates, and other organisms to detect cross-contaminated reads (see Hutter et al. 2022 for genome list), using the program BBMap from BBTools (default settings; https://jgi.doe.gov/data-and-tools/bbtools/). Next, paired-end reads are merged using BBMerge (settings: verystrict=t, k = 60, extend2=60, ecct; Bushnell et al. 2017), which also fills in missing gaps between nonoverlapping paired-end reads by assembling the missing data from the other paired-end reads. Finally, exact duplicates are also removed using “dedupe” from BBTools, removing read-pairs when both pairs were duplicated. Additionally, duplicates from the set of merged paired-end contigs were removed if they were exact duplicates or were contained within another merged set of reads.

The merged singletons and paired-end reads were next de novo assembled using the program SPADES v.3.12 (settings: careful -t, –expect-gaps, –hap-assembly; Bankevich et al. 2012), which internally runs BAYESHAMMER (Nikolenko et al. 2013) error correction on the reads. Data were assembled using several different k-mer values (21, 33, 55, 77, 99, and 127), where orthologous contigs resulting from the different k-mer assemblies were merged. We used the DIPSPADES (Safanova et al. 2015) function from this program to better assemble polymorphic exons by generating a consensus sequence from both haplotypes from orthologous regions.

The consensus haplotype contigs were then matched with Blast (settings: dc-megablast e-value < 0.001) against reference marker sequences from the *N. parkeri* genome used to design the probes, keeping only those contigs that matched uniquely to the reference probe sequences. Contigs were discarded if they did not cover at least 30% of the reference marker. Finally, we merged all discrete contigs that matched to the same reference marker, joining them together with Ns based on their match position within the marker.

The final set of matching loci was next aligned using MAFFT local pair alignment (max iterations = 1000, ep = 0.123, op = 3). Each marker was separately aligned with its corresponding reference where the probes were designed from. We screened each alignment for samples that were greater than 40% divergent from the reference sequence. Alignments were kept if they had greater than three taxa and more than 100 bp. We next separated the alignments into five data sets: 1) “Unified”, where the full-contigs set of alignments were not separated by locus type, but were kept as a single marker (i.e., introns were not trimmed off exons; UCEs were analyzed together); 2) “Exon”, each alignment was adjusted to be in an open-reading frame and trimmed to the largest reading frame that included >90% of the sequences; 3) “Intron”, the exon previously delimited was trimmed out of the full-contigs data set, and the two intronic regions were concatenated; 4) “UCE”, were separately saved and not modified; and 5) “Gene”, after separating the exons from their flanking intron sequence, exons were concatenated and grouped together in genes if they were found from the same predicted gene from the *N. parkeri* and *Xenopus tropicalis* genomes using a Blast search. Finally, the introns and UCE data sets were internally trimmed using TRIMAL (automatic1 function; Capella-Gutiérrez et al. 2009) and alignments were externally trimmed to ensure that at least 50% of the samples had sequence data at both ends. Finally, to clean up misaligned segments, we created a custom script to assess each sample in each alignment using 100 bp windows, and if that window had greater than a 40% divergence from the consensus, that sequence was replaced with Ns. Finally, we assessed missing data as the number of missing bases per alignment from samples included in the alignment (i.e., missing base-pair data) and the number of samples completely missing from an alignment (i.e., missing marker data).

### Concatenation Phylogeny

We concatenated the sets of markers described above into single alignments for maximum likelihood (ML) phylogenetic analyses. We used the maximum-likelihood method IQ-Tree v.2.0 (Nguyen et al. 2015) to estimate phylogenetic trees from the concatenated data for each molecular marker type. For these analyses, we employed models of molecular evolution identified via ModelFinder (Kalyaanamoorthy et al. 2017) built into IQ-Tree, which identified an optimal partitioning scheme and best model for each partition. We assessed support for the resulting topology using 1,000 ultrafast bootstrap replicates (Minh et al. 2013). We scripted a gene jackknifing (i.e., resampling without replacement) workflow in R to estimate topological precision across concatenated phylogenetic analyses (available in PhyloConfigR as function geneJackknife). This approach benefits from using full model selection and partitioning across data matrices, which are not computationally tractable on larger data sets.

The jackknifing approach used ML with IQ-Tree and followed the procedure: 1) alignments for the data matrix were randomly selected without replacement, where alignments that were selected up until a threshold of 200,000 bp had been reached so that each matrix that was nearly the same size in number of base pairs; 2) alignments were partitioned by codon position within exons and by marker for noncoding regions; 3) ModelFinder was used to select the best model for each partition; 4) the analysis was run across 1,000 jackknifed replicates; and 5) the 1,000 replicate trees were summarized by generating a maximum clade credibility tree using the sumtrees.py script from DENDROPY (default settings; Sukumaran and Holder 2010).

### Summary Species Trees

To perform summary species tree estimation, we use the software ASTRAL-III (Zhang et al. 2018), which conducts a summary-coalescent species tree analysis that is statistically consistent under the multi-species coalescent model (R interface for ASTRAL-III implemented in PhyloConfigR as function runAstral). As input for ASTRAL-III, individual trees for marker were needed, so we performed ML concatenation analyses on each alignment using IQ-Tree. We ran the analyses separately on the Unified, Exon, Intron, UCE, and Gene data sets. To improve accuracy, we collapsed branches that were below 10% bootstrap support, as recommended by the authors (Zhang et al. 2018). Finally, we used local branch support from quartet score frequencies to assess topological support for the coalescent trees generated by ASTRAL-III because this method out-performs multi-locus bootstrapping (Sayyari and Mirarab 2016). Local branch support was plotted as pie charts on each branch showing the quartet score frequencies (plotting implemented as function plot.Astral in PhyloConfigR).

### Site-Based Species Trees

Whereas the summary-coalescent species tree approach addresses ILS, some of the assumptions of these methods might be violated if gene trees are erroneous and if there is inter-gene recombination. To address these potential shortcomings, we use a site-based coalescent method that uses the sequence data directly and does not rely on individual gene tree estimates and incorporates substitution-rate and coalescent variance (Huang et al. 2010). We use SVDquartets that is a site-based quartet assembly heuristic because it addresses these concerns and is computationally efficient with large phylogenomic data sets (Chifman and Kubatko 2014). We used SVDquartets on all data sets evaluating quartets exhaustively (evalq = all) and used multi-locus bootstrapping (bootstrap = multilocus) with 1,000 replicates to evaluate support for each node. We used SVDquartets across each of the five data types.

### Full-Likelihood Species Trees

We used BPP (version 4.6.1; Yang 2015), which is a full-likelihood method. For BPP, and we analyzed the five data types by concatenating and formatting alignments to BPP specifications using the PhyloConfigR function generateBPP. We used the “species tree estimation” program of BPP (A01) and set up the control file with default settings except for these modifications: burnin = 10000, sampfreq = 2, nsample = 100000, and the nloci for each data set (Unified: 8679; Exon: 4328; Intron: 4197; UCE: 2762; and Gene: 1599). The theta prior was set to gamma(3, 0.004 e) and tau prior was set to gamma(3, 0.004). We set a single population for each sample in the data set as it was represented by single species. We ran each analysis twice and compared each result to ensure they converged on the same topology.

### Disentangling Incongruence

#### Branch Lengths and Support

To assess potential causes of incongruence, we tested for a relationship between branch lengths and support metrics. Branch lengths were derived from the concatenated ML analysis for each data set from the parent node, and two support metrics were calculated for each subfamily: 1) the proportion of gene trees that were monophyletic for that subfamily; and 2) the proportion of gene trees that have nodes strongly supporting (with a 90 or greater bootstrap from IQ-Tree) the monophyly for that subfamily. Finally, we used Ordinary Least Squares regression to test for a significant relationship between branch lengths and the two metrics described above. A significant positive relationship for 1) would suggest that shorter branch lengths lead to fewer gene trees supporting subfamily monophyly. A significant positive relationship for 2) would suggest that shorter branch lengths are associated with lower bootstrap support for subfamilies.

#### Data Set Filtration

Alignments that have few PIS or large amounts of missing data could potentially lead to GTEE and EGTs, which could be driving conflicting topologies between concatenation and species tree analyses. To test whether filtering out EGTs leads to consistent topologies, we apply several combinations of filtration schemes applied to individual marker alignments or tree files prior to conducting analyses in ASTRAL-III, SVDquartets, and concatenation in IQ-Tree. We did not filter for BPP analyses as this was computationally intractable with many filtered data sets. We employed concatenation on the filtered data sets to compare with the species tree methods to understand when analytical methods are consistent under filtration.

Prior to filtration, we calculated statistics for each sequence alignment used for gene tree estimation using the summarizeAlignments function from PhyloConfigR: 1) sampling, we calculated the proportion of samples included in the alignment; 2) proportion PIS, which is the number of PIS divided by the alignment length; 3) number of PIS, where we counted the number of PIS in the alignment; and 4) alignment Length, we counted the number of base-pairs in the alignment. Using the alignment statistics, we filtered the alignments under each scheme for IQ-Tree and SVDquartets using the function filterAlignments, which creates concatenated alignments for each subdata set. For ASTRAL-III, we used the alignments to filter the corresponding gene tree data set down to subsets of gene trees to estimate a new filtered species tree, using the functions filterGeneTrees from PhyloConfigR and from PhyloConfigR the function AstralRunner to run ASTRAL-III across all the filtered data sets.

We applied the following alignment filters for Astral-III and SVDquartets: 1) sampling: 0–1 at 0.05 increments (*n* = 20); 2) proportion PIS: 0–1 at 0.05 increments (*n* = 20); 3) number of PIS: 10–100 at 10 bp increments and 100–700 at 100 bp increments (*n* = 20); and 4) alignment length: 100–3,000 at 100 bp increments (*n* = 30). We selected fewer filters for concatenation because of the computational resources required, and we applied the following filters for IQ-Tree: 1) sampling: 0–1 at 0.1 increments (*n* = 10); 2) proportion PIS: 0–1 at 0.1 increments (*n* = 10); 3) number of PIS: 10, 30, 50, 70, 100, 200, 500, 700, and 1,000 (*n* = 10); and 4) alignment length: 200, 500, 700, 1,000, 1,200, 1,500, 1,700, 2,000, 2,200, and 2,500 bp (*n* = 10). For each filter, we collected the mean of each of these filtration parameters (i.e., mean alignment length of alignments >100 bp is ∼300 bp). We filtered alignments for the Unified, Exon, Intron, UCE, and Gene data sets and compared results among the different marker types.

#### Concordance Factors

To evaluate the impact of filtration on gene and site support, each analysis was evaluated using gene and site concordance factors (gCF and sCF; Minh et al. 2020). Concordance factors were calculated in IQ-Tree v. 2.0 (Minh et al. 2020). The metrics provide the relative proportion of gene trees (gCF) or sites (sCF) that can be computed for each branch in each topology. We calculated these metrics for the resulting filtered tree and used the sequence data from each filtered set of alignments to calculate gCF and sCF support for the filtered ASTRAL-III topology. To understand how filtering gene trees impacts support via gCF and sCF across our focal subfamily nodes, we used standard linear regression to test for a relationship between gCF and sCF for each focal clade. If gene trees are reflective of their underlying sites, we would expect a positive linear relationship between gCF and sCF. Additionally, we also plotted the gCF and sCF for each subfamily node without filtering (for each marker type) and with filtering to visually inspect the range of support across filtration replicates.

## Supplementary Material

evad070_Supplementary_Data

## Data Availability

All raw sequencing reads are available in the GenBank SRA (BioProject: PRJNA665754). All alignments analyzed and materials for replicating analyses are available on the Open Science Framework [https://osf.io/mybcj/ with DOI 10.17605/OSF.IO/MYBC]. The newly developed PhyloConfigR R package can be found on Carl R. Hutter's GitHub (https://github.com/chutter/PhyloConfigR), which can be used to replicate the main analyses of the paper and offers functions for filtering and creating filtered data sets. In addition, this R package offers functions to facilitate and setup analyses in ASTRAL-III, IQ-Tree, and BPP.
