# Supplementary material for: Filtration of Gene Trees From 9,000 Exons, Introns, and UCEs Disentangles Conflicting Phylogenomic Relationships in Tree Frogs (Hylidae)
Source: Genome Biol Evol. 2023 May 2;15(5):evad070. doi: 10.1093/gbe/evad070 (PMC10171231; doi:10.1093/gbe/evad070)
Supplement: evad070_Supplementary_Data [file evad070_supplementary_data.docx]

Supplementary Appendix

From:

**Filtration of Gene Trees from 9000 Exons, Introns and UCEs Disentangle Conflicting Phylogenomic Relationships in Tree Frogs (Hylidae)**

Carl R. Hutter, William Duellman

*Table of Contents:*

**Table S1.** Voucher specimens

**Table S2.** Sample raw read statistics

**Table S3**. Sample merged read statistics

**Table S4.** Sample assembled contigs statistics

**Figure S1.** Occupancy per sample for each data type
**Figure S2.** Alignments for each data type

**Figure S3.** Concatenation IQTree unfiltered tree for each data type

**Figure S4.** Gene jackknifing concatenation tree for each data type

**Figure S5.** Astral unfiltered tree for each data type

**Figure S6.** SVDQuartets unfiltered tree for each data type

**Figure S7.** BPP analysis results

**Figure S8.** Concatenation IQTree filtration results

**Figure S9.** Astral filtration results

**Figure S10.** SVDQuartets filtration results

**Figure S11.** Alternative SVDQuartets filtration results (> 50% sampling)

**Table S1.** Voucher specimen field and catalogue numbers used in this study.

| **Species** | **Field number** | **Catalogue number** |
| --- | --- | --- |
| *Acris blanchardi* | DSM 2012 | KU 337016 |
| *Callimedusa tomopterna* | WED 55506 | KU 295429 |
| *Dendropsophus koechlini* | WED 57879 | KU 215222 |
| *Dendropsophus leucophyllatus* | WED 59288 | KU 215276 |
| *Dendropsophus parviceps* | MZUTI 1357 | MZUTI 1357 |
| *Dryophytes cinerea* | WED 56355 | KU 297358 |
| *Dryophytes walkeri* | MVZ 263408 | MVZ 263408 |
| *Hyla sarda* | WED 54544 | KU 207375 |
| *Hyliola cadaverina* | WED 54461 | KU 207470 |
| *Hyloscirtus phyllognathus* | WED 58378 | KU 212118 |
| *Hyloscirtus staufferorum* | LAC 2153 | KU 217694 |
| *Hypsiboas boans* | WED 57877 | KU 215193 |
| *Litoria infrafrenata* | SLT 771 | KU 345125 |
| *Lysapsus laevis* | CAS 257655 | CAS 257655 |
| *Osteocephalus taurinus* | WED 55452 | KU 205406 |
| *Plectrohyla quecchi* | MVZ 251534 | MVZ 251534 |
| *Pseudacris triseriata* | BLO 6 | KU 332199 |
| *Pseudis paradoxus* | CAS 245053 | CAS 245053 |
| *Ptychohyla salvadorensis* | EBG 518 | KU 289957 |
| *Scarthyla goinorum* | WED 58246 | KU 205914 |
| *Scinax garbei* | LAC 1787 | KU 217703 |
| *Scinax ruber* | WED 56140 | KU 207619 |
| *Smilisca phaeota* | LAC 2299 | KU 217762 |
| *Sphaenorhynchus lacteus* | WED 54090 | KU 202766 |
| *Trachycephalus jordani* | WED 53658 | KU 202747 |
| *Trachycephalus typhonius* | WED 55450 | KU 205418 |

**Table S2.** A summary of the raw read data from sequencing is shown for each sample. Pre-QC reads are those after demultiplexing the lane, while Post-QC are reads after adaptor contamination, external contamination, and duplicate removal.

|  | ***Pre-QC*** | | ***Post-QC*** | |
| --- | --- | --- | --- | --- |
| **Sample** | **Raw reads** | ***N* mb** | **Raw reads** | ***N* mb** |
| *Acris blanchardi* KU 337016 | 6,408,006 | 968 | 5,378,070 | 793 |
| *Hyloscirtus staufferorum* KU 217694 | 5,132,106 | 775 | 4,497,706 | 668 |
| *Dendropsophus koechlini* KU 215222 | 7,444,634 | 1,124 | 788,596 | 117 |
| *Dendropsophus leucophyllatus* KU 215276 | 7,317,812 | 1,105 | 5,553,226 | 820 |
| *Dendropsophus parviceps* MZUTI 1357 | 6,544,832 | 988 | 5,488,160 | 812 |
| *Dryophytes cinerea* KU 297358 | 5,987,516 | 904 | 4,851,916 | 710 |
| *Hyla sarda* KU 207375 | 5,274,930 | 797 | 4,325,352 | 640 |
| *Dryophytes walkeri* MVZ 263408 | 4,715,914 | 712 | 3,870,574 | 562 |
| *Hyliola cadaverina* KU 207470 | 9,498,128 | 1,434 | 2,217,986 | 330 |
| *Hyloscirtus phyllognathus* KU 212118 | 6,174,074 | 932 | 5,489,806 | 820 |
| *Hypsiboas boans* KU 215193 | 6,339,332 | 957 | 5,769,474 | 860 |
| *Lysapsus laevis* CAS 257655 | 9,489,440 | 1,433 | 7,233,788 | 1,055 |
| *Litoria infrafrenata* KU 345125 | 13,151,808 | 1,986 | 10,950,766 | 1,599 |
| *Osteocephalus taurinus* KU 205406 | 2,941,830 | 444 | 2,522,728 | 376 |
| *Callimedusa* *tomopterna* KU 295429 | 7,762,348 | 1,172 | 6,370,522 | 931 |
| *Trachycephalus typhonius* KU 205418 | 7,242,892 | 1,094 | 6,156,310 | 909 |
| *Plectrohyla quecchi* MVZ 251534 | 4,379,924 | 661 | 3,513,244 | 518 |
| *Pseudacris triseriata* KU 332199 | 7,708,084 | 1,164 | 5,790,524 | 854 |
| *Pseudis paradoxus* CAS 245053 | 19,228,002 | 2,903 | 15,311,020 | 2,242 |
| *Ptychohyla salvadorensis* KU 289957 | 7,334,260 | 1,107 | 5,736,298 | 844 |
| *Scarthyla goinorum* KU 205914 | 8,031,776 | 1,213 | 6,434,072 | 945 |
| *Scinax garbei* KU 217703 | 8,000,090 | 1,208 | 6,002,556 | 879 |
| *Scinax ruber* KU 207619 | 13,131,344 | 1,983 | 10,898,572 | 1,604 |
| *Smilisca phaeota* KU 217762 | 5,066,046 | 765 | 4,165,110 | 615 |
| *Sphaenorhynchus lacteus* KU 202766 | 7,098,854 | 1,072 | 5,782,846 | 853 |
| *Trachycephalus jordani* KU 202747 | 2,777,240 | 419 | 2,393,336 | 354 |

**Table S3.** A summary of the paired-end sequencing data before and after merging paired-end read data.

|  | ***Before merge*** | ***After merge*** | |
| --- | --- | --- | --- |
| **Sample** | ***N* pairs** | ***N* pairs** | **Merged reads** |
| *Acris blanchardi* KU 337016 | 2,689,035 | 1,559,519 | 344,278 |
| *Hyloscirtus staufferorum* KU 217694 | 2,248,853 | 1,152,788 | 320,572 |
| *Dendropsophus koechlini* KU 215222 | 394,298 | 222,977 | 87,772 |
| *Dendropsophus leucophyllatus* KU 215276 | 2,776,613 | 1,786,740 | 379,365 |
| *Dendropsophus parviceps* MZUTI 1357 | 2,744,080 | 1,556,263 | 388,963 |
| *Dryophytes cinerea* KU 297358 | 2,425,958 | 1,466,410 | 295,877 |
| *Hyla sarda* KU 207375 | 2,162,676 | 1,323,389 | 270,385 |
| *Dryophytes walkeri* MVZ 263408 | 1,935,287 | 1,131,590 | 300,815 |
| *Hyliola cadaverina* KU 207470 | 1,108,993 | 633,700 | 184,749 |
| *Hyloscirtus phyllognathus* KU 212118 | 2,744,903 | 1,440,434 | 335,004 |
| *Hypsiboas boans* KU 215193 | 2,884,737 | 1,373,755 | 438,400 |
| *Lysapsus laevis* CAS 257655 | 3,616,894 | 2,148,454 | 317,771 |
| *Litoria infrafrenata* KU 345125 | 5,475,383 | 2,868,889 | 519,747 |
| *Osteocephalus taurinus* KU 205406 | 1,261,364 | 740,659 | 202,547 |
| *Callimedusa* *tomopterna* KU 295429 | 3,185,261 | 2,095,809 | 426,367 |
| *Trachycephalus typhonius* KU 205418 | 3,078,155 | 1,804,700 | 378,968 |
| *Plectrohyla quecchi* MVZ 251534 | 1,756,622 | 1,124,779 | 198,068 |
| *Pseudacris triseriata* KU 332199 | 2,895,262 | 1,951,931 | 356,019 |
| *Pseudis paradoxus* CAS 245053 | 7,655,510 | 4,106,396 | 755,347 |
| *Ptychohyla salvadorensis* KU 289957 | 2,868,149 | 1,623,708 | 310,518 |
| *Scarthyla goinorum* KU 205914 | 3,217,036 | 2,037,607 | 360,609 |
| *Scinax garbei* KU 217703 | 3,001,278 | 2,029,662 | 338,365 |
| *Scinax ruber* KU 207619 | 5,449,286 | 3,227,210 | 672,407 |
| *Smilisca phaeota* KU 217762 | 2,082,555 | 1,159,312 | 252,275 |
| *Sphaenorhynchus lacteus* KU 202766 | 2,891,423 | 1,873,652 | 404,990 |
| *Trachycephalus jordani* KU 202747 | 1,196,668 | 645,911 | 197,041 |

**Table S4.** A summary of the assembled contigs and their length after assembly with SPADES.

|  |  | ***Length statistics*** | | | |
| --- | --- | --- | --- | --- | --- |
| **Sample** | **N contigs** | **Mean** | **Min** | **Max** | **sd** |
| *Acris blanchardi* KU 337016 | 20,724 | 794 | 130 | 11,616 | 422 |
| *Hyloscirtus staufferorum* KU 217694 | 13,327 | 967 | 129 | 16,301 | 645 |
| *Dendropsophus koechlini* KU 215222 | 5,378 | 807 | 133 | 8,437 | 455 |
| *Dendropsophus leucophyllatus* KU 215276 | 18,106 | 878 | 131 | 20,515 | 555 |
| *Dendropsophus parviceps* MZUTI 1357 | 18,099 | 842 | 130 | 9,879 | 416 |
| *Dryophytes cinerea* KU 297358 | 14,967 | 859 | 135 | 15,848 | 493 |
| *Hyla sarda* KU 207375 | 16,279 | 833 | 130 | 6,879 | 409 |
| *Dryophytes walkeri* MVZ 263408 | 11,016 | 778 | 129 | 8,458 | 477 |
| *Hyliola cadaverina* KU 207470 | 11,951 | 810 | 129 | 10,589 | 461 |
| *Hyloscirtus phyllognathus* KU 212118 | 16,068 | 945 | 129 | 20,664 | 566 |
| *Hypsiboas boans* KU 215193 | 16,015 | 912 | 130 | 22,652 | 621 |
| *Lysapsus laevis* CAS 257655 | 22,659 | 876 | 131 | 7,962 | 453 |
| *Litoria infrafrenata* KU 345125 | 25,788 | 892 | 129 | 14,509 | 494 |
| *Osteocephalus taurinus* KU 205406 | 12,079 | 880 | 133 | 16,243 | 499 |
| *Callimedusa* *tomopterna* KU 295429 | 16,765 | 866 | 129 | 15,847 | 591 |
| *Trachycephalus typhonius* KU 205418 | 21,179 | 884 | 129 | 16,372 | 459 |
| *Plectrohyla quecchi* MVZ 251534 | 13,359 | 911 | 130 | 16,156 | 524 |
| *Pseudacris triseriata* KU 332199 | 21,198 | 841 | 129 | 10,805 | 505 |
| *Pseudis paradoxus* CAS 245053 | 37,957 | 873 | 131 | 16,051 | 509 |
| *Ptychohyla salvadorensis* KU 289957 | 18,486 | 866 | 132 | 10,370 | 511 |
| *Scarthyla goinorum* KU 205914 | 20,396 | 892 | 131 | 16,325 | 534 |
| *Scinax garbei* KU 217703 | 19,751 | 877 | 128 | 12,842 | 533 |
| *Scinax ruber* KU 207619 | 30,772 | 893 | 129 | 18,322 | 531 |
| *Smilisca phaeota* KU 217762 | 14,623 | 840 | 130 | 10,730 | 472 |
| *Sphaenorhynchus lacteus* KU 202766 | 18,014 | 841 | 131 | 16,054 | 487 |
| *Trachycephalus jordani* KU 202747 | 11,231 | 894 | 129 | 17,312 | 498 |

**Figure S1.** The number of markers used for alignments from the five data types (Unified also called Unpartitioned, Exon, Intron, Gene, UCE) are shown for each sample.

**Figure S2.** Alignment statistics for the five data types. Alignments are summarized as: (A) the number of alignments for each data type and (B) the number of alignments from each size class for each data type.

**Figure S3.1** Concatenation tree estimated using the unfiltered alignments from the Unified (target contigs that include exons + introns together in one alignment) dataset. All nodes have 100 ultra-fast bootstrap support.

**Figure S3.2** Concatenation tree estimated using the unfiltered alignments from the Exon dataset. All nodes have 100 ultra-fast bootstrap support.

**Figure S3.3** Concatenation tree estimated using the unfiltered alignments from the Intron dataset. All nodes have 100 ultra-fast bootstrap support.

**Figure S3.4** Concatenation tree estimated using the unfiltered alignments from the UCE dataset. All nodes have 100 ultra-fast bootstrap support.

**Figure S3.5** Concatenation tree estimated using the unfiltered alignments from the Gene (exons from the same gene are concatenated together) dataset. All nodes have 100 ultra-fast bootstrap support.

**Figure S4.1** Gene-jackknifing summary tree estimated using the unfiltered alignments from the Unified (target contigs that include exons + introns together in one alignment) dataset. Pie charts at nodes show the proportion of bootstrap replicates that strongly support that node.

**Figure S4.2** Gene-jackknifing summary tree estimated using the unfiltered alignments from the Exon dataset. Pie charts at nodes show the proportion of bootstrap replicates that strongly support that node.

**Figure S4.3** Gene-jackknifing summary tree estimated using the unfiltered alignments from the Intron dataset. Pie charts at nodes show the proportion of bootstrap replicates that strongly support that node.

**Figure S4.4** Gene-jackknifing summary tree estimated using the unfiltered alignments from the UCE dataset. Pie charts at nodes show the proportion of bootstrap replicates that strongly support that node.

**Figure S4.5** Gene-jackknifing summary tree estimated using the unfiltered alignments from the Gene (exons from the same gene are concatenated together) dataset. Pie charts at nodes show the proportion of bootstrap replicates that strongly support that node.

**Figure S5.1** Astral species tree summarized from the unfiltered gene trees that includes all non-overlapping trees. This includes the gene trees from the Exon, Intron, UCE, and Gene datasets, while excluding the individual exons from the Exon dataset concatenated for the Gene dataset. Pie charts on branches show the proportion of gene trees that support that quadripartition.

**Figure S5.2** Astral species tree summarized from the unfiltered gene trees from the Unified (target contigs that include exons + introns together in one alignment) dataset. Pie charts on branches show the proportion of gene trees that support that quadripartition.

**Figure S5.3** Astral species tree summarized from the unfiltered gene trees from the Exon dataset. Pie charts on branches show the proportion of gene trees that support that quadripartition.

**Figure S5.4** Astral species tree summarized from the unfiltered gene trees from the Intron dataset. Pie charts on branches show the proportion of gene trees that support that quadripartition.

**Figure S5.5** Astral species tree summarized from the unfiltered gene trees from the UCE dataset. Pie charts on branches show the proportion of gene trees that support that quadripartition.

**Figure S5.6** Astral species tree summarized from the unfiltered gene trees from the Gene (exons from the same gene are concatenated together) dataset. Pie charts on branches show the proportion of gene trees that support that quadripartition.

**Figure S6.1** SVDquartets species tree estimated from the Unified dataset, which included alignments from all datatypes, where exons and introns were not separated, and flanking regions remained for all alignments. All nodes have greater than 95 bootstrap unless indicated with a colored dot at the node.

**Figure S6.2** SVDQuartets species tree estimated using the concatenated unfiltered alignments from the Exon dataset. All nodes have greater than 95 bootstrap unless indicated with a colored dot at the node.

**Figure S6.3** SVDQuartets species tree estimated using the concatenated unfiltered alignments from the Intron dataset. All nodes have greater than 95 bootstrap unless indicated with a colored dot at the node.

**Figure S6.4** SVDQuartets species tree estimated using the concatenated unfiltered alignments from the UCE dataset. All nodes have greater than 95 bootstrap unless indicated with a colored dot at the node.

**Figure S6.5** SVDQuartets species tree estimated using the concatenated unfiltered alignments from the Gene (exons from the same gene are concatenated together) dataset. All nodes have greater than 95 bootstrap unless indicated with a colored dot at the node.

**Figure S7.1** BPP species tree estimated from the Unified dataset, which included alignments from all datatypes, where exons and introns were not separated, and flanking regions remained for all alignments. Support values from BPP are shown at each node and branch lengths are equal and plotting as a cladogram.

**Figure S7.2** BPP species tree estimated using the concatenated unfiltered alignments from the Exon dataset. Support values from BPP are shown at each node and branch lengths are equal and plotting as a cladogram.

**Figure S7.3** BPP species tree estimated using the concatenated unfiltered alignments from the Intron dataset. Support values from BPP are shown at each node and branch lengths are equal and plotting as a cladogram.

**Figure S7.4** BPP species tree estimated using the concatenated unfiltered alignments from the UCE dataset. Support values from BPP are shown at each node and branch lengths are equal and plotting as a cladogram.

**Figure S7.5** BPP species tree estimated using the concatenated unfiltered alignments from the Gene (exons from the same gene are concatenated together) dataset. Support values from BPP are shown at each node and branch lengths are equal and plotting as a cladogram.

**Figure S8** Expanded alignment filtering phylogenetic results from IQTree across the Unpartitioned, Exons, Introns, UCEs and Genes datasets. The four filters used were (A) individual marker alignments were filtered by the proportion of sampled taxa in that alignment; (B) the proportion of sites in an alignment that were informative (number of informative sites divided by alignment length); (C) the number of informative sites in an alignment; and (D) the base-pair length of the alignment. Support was measured using the bootstrap and was categorized as: Strong: greater 95; Moderate: 70-90; Weak: less than 70.

**Figure S9** Expanded gene and alignment filtering phylogenetic results from ASTRAL-III across the Unpartitioned, Exons, Introns, UCEs and Genes datasets. The four filters used were (A) individual marker alignments were filtered by the proportion of sampled taxa in that alignment; (B) the proportion of sites in an alignment that were informative (number of informative sites divided by alignment length); (C) the number of informative sites in an alignment; and (D) the base-pair length of the alignment. Support was measured using the posterior probability and was categorized as: Strong: greater than 0.95; Moderate: 0.70-0.95; Weak: less than 0.70.

**Figure S10** Expanded gene and alignment filtering phylogenetic results from SVDQuartets across the Unpartitioned, Exons, Introns, UCEs and Genes datasets. The four filters used were (A) individual marker alignments were filtered by the proportion of sampled taxa in that alignment; (B) the proportion of sites in an alignment that were informative (number of informative sites divided by alignment length); (C) the number of informative sites in an alignment; and (D) the base-pair length of the alignment. Support was measured using the bootstrap and was categorized as: Strong: greater 95; Moderate: 70-90; Weak: less than 70.

**Figure S11** Alternative SVDQuartets analyses (see Figure S8) where each alignment is also filtered at 50% or greater for taxon sampling. The four filters used were (A) individual marker alignments were filtered by the proportion of sampled taxa in that alignment; (B) the proportion of sites in an alignment that were informative (number of informative sites divided by alignment length); (C) the number of informative sites in an alignment; and (D) the base-pair length of the alignment. Support was measured using the bootstrap and was categorized as: Strong: greater 95; Moderate: 70-90; Weak: less than 70.
